# CD109, a TGF-β co-receptor, attenuates extracellular matrix production in scleroderma skin fibroblasts

**DOI:** 10.1186/ar3877

**Published:** 2012-06-13

**Authors:** Xiao-Yong Man, Kenneth W Finnson, Murray Baron, Anie Philip

**Affiliations:** 1 Division of Plastic Surgery, Department of Surgery, McGill University, Montreal General Hospital, 1650 Cedar Avenue, Montreal, H3G 1A4,Canada; 2 Department of Rheumatology, McGill University, Jewish General Hospital, 3755 Cote St Catherine Road, Montreal, H3T 1E2,Canada

## Abstract

**Introduction:**

Scleroderma or systemic sclerosis (SSc) is a complex connective tissue disease characterized by fibrosis of skin and internal organs. Transforming growth factor beta (TGF-β) plays a key role in the pathogenesis of SSc fibrosis. We have previously identified CD109 as a novel TGF-β co-receptor that inhibits TGF-β signaling. The aim of the present study was to determine the role of CD109 in regulating extracellular matrix (ECM) production in human SSc skin fibroblasts.

**Methods:**

CD109 expression was determined in skin tissue and cultured skin fibroblasts of SSc patients and normal healthy subjects, using immunofluorescence, western blot and RT-PCR. The effect of CD109 on ECM synthesis was determined by blocking CD109 expression using CD109-specific siRNA or addition of recombinant CD109 protein, and analyzing the expression of ECM components by western blot.

**Results:**

The expression of CD109 proteinis markedly increased in SSc skin tissue *in vivo *and in SSc skin fibroblasts *in vitro *as compared to their normal counterparts. Importantly, both SSc and normal skin fibroblasts transfected with CD109-specific siRNA display increased fibronectin, collagen type I and CCN2 protein levels and enhanced Smad2/3 phosphorylation compared with control siRNA transfectants. Furthermore, addition of recombinant CD109 protein decreases TGF-β_1_-induced fibronectin, collagen type I and CCN2 levels in SSc and normal fibroblasts.

**Conclusion:**

The upregulation of CD109 protein in SSc may represent an adaptation or consequence of aberrant TGF-β signaling in SSc. Our finding that CD109 is able to decrease excessive ECM production in SSc fibroblasts suggest that this molecule has potential therapeutic value for the treatment of SSc.

## Introduction

Scleroderma or systemic sclerosis (SSc) is a complex connective tissue disorder characterized by autoimmunity, vasculopathy and progressive fibrosis of skin and internal organs [1-3]. SSc is commonly classified into two major clinical subsets, diffuse SSc and limited SSc, based largely on the degree of skin involvement [4]. Although there are a number of disease characteristics that differentiate between these two groups, both share the common clinical hallmark of fibrosis - characterized by excessive extracellular matrix (ECM) production, leading to disruption of normal tissue architecture and eventually organ failure [5]. Although much progress has been made in understanding the molecular mechanisms underlying the pathophysiology of SSc [2,6,7], there are currently no therapies to halt the fibrotic process or to slow progression of the disease [5,8,9].

Transforming growth factor beta (TGF-β) is a multifunctional cytokine that regulates cell proliferation, cell differentiation and ECM production [10-12]. TGF-β is the most potent profibrotic cytokine known and is thought to play a key role in SSc pathogenesis [2,6,13,14]. Cultured SSc fibroblasts display constitutively elevated ECM synthesis, which has been attributed to aberrant activation of autocrine TGF-β signaling [15,16]. Some studies have demonstrated increased TGF-β receptor levels in SSc fibroblasts [17-19] that might contribute to activation of autocrine TGF-β signaling [16]. However, these findings have not been universally reproduced [20,21], emphasizing the need for further investigation.

TGF-β signaling is transduced by a pair of transmembrane serine/threonine kinases known as the TGF-β type I and type II receptors [22]. TGF-β binds the TGF-β type II receptor, which then recruits and phosphorylates the TGF-β type I receptor resulting in activation of TGF-β type I receptor kinase activity [23,24]. The TGF-β type I receptor propagates the signal by phosphorylating intracellular Smad2 and Smad3 proteins, which form a complex with Smad4. The Smad complexes then translocate to the nucleus where they interact with various co-activators, co-repressors and transcription factors to regulate target gene expression [12,25,26]. Important TGF-β target genes relevant to fibrotic progression in SSc include ECM proteins such as fibronectin and collagen type I and the matricellular protein CCN2 [2,27].

CD109 is a 180 kDa glycosylphosphatidylinositol-anchored protein belonging to the α_2_-macroglobulin/complement superfamily [28,29]. Although CD109 is expressed in a variety of cell types and its expression is altered in many types of cancer, the function of this protein is poorly understood [28-35]. We have recently identified CD109 as a TGF-β co-receptor and inhibitor of TGF-β signaling in human keratinocytes [36,37]. The purpose of the current study was to determine whether CD109 expression is altered in SSc skin and whether CD109 may decrease the uncontrolled production of ECM proteins by SSc skin fibroblasts.

## Materials and methods

### Human subjects

Nineteen patients diagnosed with SSc (17 females, two males; Table 1) and nine normal healthy subjects (seven females and two males aged 35 to 55 years) were studied. Diagnosis of SSc was performed according to the classification criteria of the American College of Rheumatology [38]. Punch biopsies were obtained from the dorsal forearm of subjects with informed consent. Biopsies were divided into two parts, with one part placed in 10% buffered formalin for immunohistochemistry and the other part in DMEM for fibroblast isolation. This study was approved by the Institutional Review Committee of McGill University.

**Table 1:** Patient information

Patient	Gender	Age (years)	Modified Rodnan Skin Score	Type of systemic sclerosis	Disease duration (years)	CD109 intensity (arbitrary units)^a ^
S1	Female	49	1	Limited	2.93	1.15
S2	Female	62	9	Limited	4.72	0.53
S3	Female	56	6	Diffuse	5.46	0.37
S4	Female	76	10	Limited	8.87	0.74
S5	Female	53	2	Limited	2.38	0.89
S6	Female	63	5	Limited	11.54	1.02
S7	Female	58	33	Diffuse	1.51	0.65
S8	Female	51	31	Diffuse	13.63	0.92
S9	Female	66	4	Limited	5.62	1.39
S10	Female	45	41	Diffuse	5.62	0.29
S11	Female	55	29	Diffuse	12.60	0.58
S12	Female	53	2	Limited	2.45	1.28
S13	Female	70	7	Diffuse	17.45	2.42
S14	Female	68	10	Diffuse	5.68	1.47
S15	Female	63	2	Limited	28.87	0.35
S16	Male	69	4	Limited	1.66	1.45
S17	Male	77	2	Limited	13.46	ND
S18	Female	49	0	Limited	6.00	ND
S19	Female	60	1	Limited	23.75	ND

Modified Rodnan Skin Score from [54]. ND, not determined. ^a^See Figure 2A, B.

### Cell isolation and culture

Biopsies were incubated in 0.5% dispase (Invitrogen, Carlsbad, CA, USA) overnight at 4°C, and the dermis was separated from the epidermis. The epidermis was incubated with 0.25% trypsin (Invitrogen) for 20 minutes and the released keratinocytes were cultured in keratinocyte serum-free media (Invitrogen). The dermis was incubated with 0.1% collagenase (Invitrogen) overnight and the released fibroblasts were cultured in DMEM containing 10% fetal bovine serum. Experiments were performed using fibroblasts between passages 3 and 6. Fibroblasts were serum-starved for 24 hours and treated with TGF-β_1 _(Genzyme Corporation, Framingham, MA, USA) without or with recombinant CD109 (R&D Systems Inc., Minneapolis, MN, USA). Cell lysates were prepared and stored at -80°C until further analysis.

### Immunohistochemistry

SSc and normal skin biopsies were fixed in formalin, embedded in paraffin and cut into 4 μm serial sections using a microtome. SSc and normal skin fibroblasts cultured on glass coverslips were fixed with 4% paraformaldehyde. Tissue sections and cells were deparaffinized and rehydrated, and antigen retrieval was performed by heating at 95°C for 20 minutes in sodium citrate buffer (10 mM, pH 8.5). The samples were then permeabilized with PBS containing 0.1% Triton X-100. Blocking was performed with 10% normal rabbit serum for 1 hour at room temperature. Sections were then incubated with a mouse anti-human CD109 antibody (R&D Systems Inc.) overnight at 4°C, followed by an Alexa Fluor 488-conjugated rabbit anti-mouse secondary antibody (AF-488; Invitrogen) for 2 hours at room temperature. Immunofluorescent images were obtained using a fluorescence microscope (Olympus B202; Carson Group Inc., Markham, ON, Canada) with a digital camera. Alternatively, sections incubated with the anti-human CD109 antibody as above were detected with a horseradish peroxidase-conjugated rabbit anti-mouse secondary antibody followed by diaminobenzidine staining.

### Reverse transcription-polymerase chain reaction

Total RNA was extracted from SSc and normal skin fibroblasts using the RNeasy Mini Kit (Qiagen, Mississauga, ON, Canada) and reverse transcribed using MMLV reverse transcriptase with oligo-dT as primer. PCR was performed using CD109 (forward primer, 5'-GCCTTTGATTTAGATGTTGCTGTA-3'; reverse primer, 5'-TATTCCACTTTCTTCACTGTCTCG-3'; product length 188 bp) and GAPDH (forward primer, 5'-GGGGAGCCAAAAGGGTCATCATCT-3'; and reverse primer, 5'-TTGGCCAGGGGTGCTAAG-3'; product length 145 bp) primers and Taq DNA polymerase (Invitrogen). After denaturation at 95°C for 5 minutes, the reaction was performed for 25 cycles at 95°C for 30 seconds, 59°C for 30 seconds and 72°C for 30 seconds. The PCR products were separated in 1.5% agarose gel and visualized by ethidium bromide staining.

Real-time PCR was performed using the Bio-Rad CFX96 Real-Time System using the RT^2 ^SYBR Green qPCR Master Mix (Qiagen Inc., Mississauga, ON, Canada) with CD109-specific primers (product size 147 bp, catalogue number PPH10537A; SABiosciences) and GAPDH-specific primers (forward primer, 5'-AAGATCATCAGCAATGCCTCCTG-3'; and reverse primer, 5'-TGACCTTGCCCACAGCCTT-3'; product length 228 bp). PCR was performed with an initial denaturation step for 10 minutes at 95°C followed by 40 cycles at 95°C for 15 seconds, 55°C for 30 seconds and 72°C for 30 seconds. Data were analyzed using the ΔC_t _method and are presented as the fold-change in ΔC_t_.

### siRNA transfection

SSc and normal skin fibroblasts were transfected with CD109-specific siRNA (Stealth™; Invitrogen) or control siRNA using the TransIT-LT1 transfection reagent (Mirus Bio LLC, Madison, WI, USA). Cell lysates were prepared 48 hours later and were analyzed by western blot.

### Western blot

Cell lysates were prepared in RIPA buffer (50 mM Tris-HCl, 1% NP-40, 0.25% sodium deoxycholate, 150 mM NaCl, 1 mM ethylenediamine tetraacetic acid, 1 mM phenylmethylsulfonyl fluoride, 1 mM Na_3_VO_4_, 1 mM NaF and Roche complete Protease Inhibitor Cocktail; Roche Applied Biosciences, Laval, QC, Canada), and protein concentrations were determined. Cell lysates (15 μg per lane) were separated by SDS-PAGE and transferred onto nitrocellulose membranes (Millipore, Bedford, MA, USA). Following blocking with 5% nonfat dry milk in Tris-buffered saline-Tween at room temperature for 1 hour, membranes were incubated overnight with antibodies against CD109 (R&D Systems Inc.), pSmad2, pSmad3 and Smad2 (all from Cell Signaling Technology, Danvers, MA, USA), collagen type I (Abcam, Cambridge, MA, USA), fibronectin (BD Biosciences, Mississauga, ON, Canada) and CCN2, Smad3 and β-actin (all from Santa Cruz Biotechnology, Santa Cruz, CA, USA) followed by incubation with a horseradish peroxidase-conjugated secondary antibody. Immunoblots were developed with enhanced-chemiluminescence reagents (GE Healthcare, Baie d'Urfe, QC, Canada). Densitometric analysis was performed using Gel-Pro Analyzer software V4.0 (Media Cybernetics, Bethesda, MD, USA) and statistical analysis was performed by one-way analysis of variance followed by an all-pairwise multiple comparison procedure (Holm-Sidak method) with an overall significance level of 0.05.

## Results

### CD109 protein expression is increased in SSc skin compared with normal skin *in vivo*

**Figure 1:**
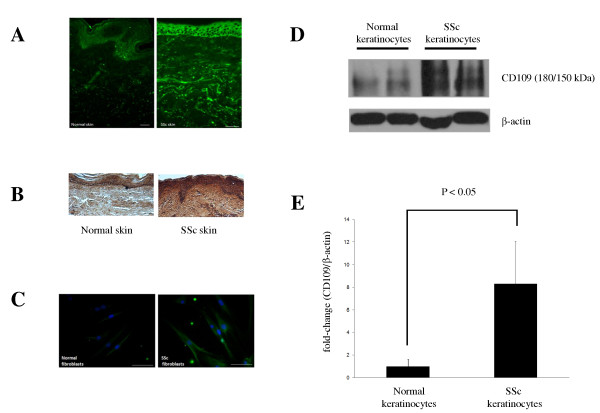
**CD109 protein expression is increased in systemic sclerosis skin and in cultured systemic sclerosis skin fibroblasts**. **(A, B) **Immunohistochemistry: systemic sclerosis (SSc) (Patients S1 and S19 in Table 1) and normal (controls N1 and N9) skin sections were analyzed by immunohistochemistry using an anti-CD109 antibody followed by detection with (A) Alexa Fluor 488-conjugated secondary antibody (Patient S1 and control N1) or (B) horseradish peroxidase-conjugated secondary antibody (Patient S19 and control N9) followed by diaminobenzidine staining, as described in Materials and methods. **(C) **Immunocytochemistry: SSc (Patient S1) and normal (control N1) skin fibroblasts were grown in culture and were analyzed by immunocytochemistry using an anti-CD109 antibody followed by detection with an Alexa Fluor 488-conjugated secondary antibody, as described in Materials and methods. Immunofluorescence microscopy was done using Olympus B202 (Carson Group Inc., Markham, ON, Canada). Images captured with a digital camera (DC-330; Dage-MTI Inc., Michigan City, IN, USA). Scale bar: 50 μM. Results presented are representative of three independent experiments. **(D, E) **Western blot: cell lysates prepared from cultured SSc and normal keratinocytes were analyzed by western blot using an anti-CD109 antibody. The membrane was reprobed with an anti-β-actin antibody to confirm equal protein loading (D). Densitometric analysis of CD109 protein levels (180 kDa band) in the cell lysates of SSc (*n *= 6) and normal (*n *= 6) keratinocytes was performed using β-actin as a loading control (*P *< 0.05).

We have previously demonstrated that CD109 is expressed *in vitro *in normal human keratinocytes [37,39,40] and fibroblasts [41,42]. Here we examined CD109 expression in SSc and normal skin tissue by immunohistochemistry. Figure 1A shows that CD109 protein is detected in SSc skin (right panel) and normal skin (left panel). Notably, the expression of CD109 protein is markedly increased in both the dermis and epidermis of SSc skin compared with those of normal skin, as detected by a more intense immunofluorescent signal using an Alexa Fluor 488-conjugated secondary antibody (Figure 1A). Similar results were obtained by immunostaining using a horseradish peroxidase-conjugated secondary antibody with diaminobenzidine as a substrate (Figure 1B).

### CD109 protein expression is increased in SSc skin cells (fibroblasts and keratinocytes) compared with normal skin cells *in vitro*

Fibroblasts are the main cell type responsible for excessive ECM production in SSc [13], and cultured SSc fibroblasts continue to express elevated ECM production compared with normal fibroblasts [16]. We therefore analyzed CD109 expression in cultured SSc and normal skin fibroblasts, using immunofluorescence detection. The results shown in Figure 1C demonstrate that CD109 protein is detected in both SSc and normal skin fibroblasts in culture and is localized to the plasma membrane, with diffuse staining in the cytoplasm. Although the cytoplasmic staining may represent newly synthesized CD109 protein and/or CD109 protein that has undergone internalization, the method used does not allow us to precisely define the localization of CD109 in these cells. Importantly, SSc skin fibroblasts display increased CD109 protein levels compared with normal skin fibroblasts.

Because CD109 protein levels are also increased in SSc epidermis compared with normal epidermis (Figure 1A, B), we sought to determine CD109 protein expression in cultured SSc and normal keratinocytes. Figure 1D shows that CD109 protein levels are markedly increased in cell lysates from SSc keratinocytes compared with normal keratinocytes as determined by western blot (upper panel). Reprobing the membrane with an anti-β-actin antibody confirms that equal amounts of protein were loaded in each lane. Densitometric analysis of CD109 protein levels (180 kDa band) in the cell lysates of SSc keratinocytes (*n *= 6) and normal keratinocytes (*n *= 6) was then performed using β-actin as a loading control. The results indicate that SSc keratinocytes display significantly (*P *< 0.05) higher CD109 protein levels compared with normal keratinocytes (Figure 1E).

### Elevated CD109 protein expression is not associated with an increase in CD109 mRNA expression in SSc skin fibroblasts

**Figure 2:**
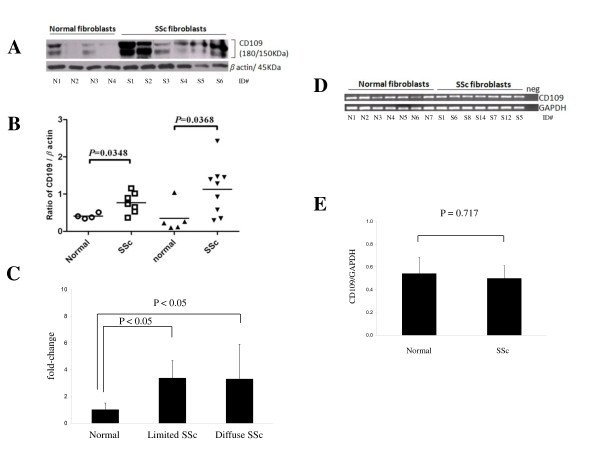
**CD109 protein, but not mRNA, expression is increased in systemic sclerosis skin fibroblasts**. **(A) **Cell lysates prepared from systemic sclerosis (SSc) (in = 6, Patients S1 to S6) and normal (*n *= 4, controls N1 to N4) skin fibroblasts were analyzed by western blot using an anti-CD109 antibody, as described in Materials and methods. The membrane was reprobed with an anti-β-actin antibody to confirm that equal amounts of protein were loaded in each lane. **(B) **Densitometric analysis of CD109 protein levels (180 kDa band) in the cell lysates of SSc and normal skin fibroblasts was performed using β-actin as a loading control. Results from two independent experiments corresponding to skin fibroblasts from two different randomly selected groups of SSC patients versus normal subjects are shown: Experiment 1, normal (*n *= 4, controls N1 to N4) and SSc (*n *= 7, Patients S1 to S7); Experiment 2, normal (*n *= 5, controls N5 to N9) and SSc (*n *= 9, Patients S8 to S16). *P *values denote statistical differences of the means between normal and SSc fibroblasts using Student's *t *test. **(C) **Data presented in (B) obtained from 16 SSc patients (nine with limited SSc and seven with diffuse SSc) and nine normal healthy controls were reorganized into normal control, limited SSc and diffuse SSc groups and were reanalyzed. One data point (outlier) from the control group (value = 1.04) was removed. Fold-change in the ratio of CD109/β actin in the limited SSc and diffuse SSc groups compared with normal controls is shown. One-way analysis of variance analysis showed a statistically significant difference (*P *= 0.0127); Holm-Sidak analysis overall significance level = 0.05. Significant differences observed are indicated. **(D) **Total RNA extracted from SSc (*n *= 7, Patients S1, S6, S8, S14, S7, S12 and S5) and normal (*n *= 7, controls N1 to N7) skin fibroblasts was analyzed by RT-PCR using human CD109-specific (top panel) or GAPDH-specific (bottom panel) oligonucleotide primers. PCR products were resolved by agarose gel (1.5%) electrophoresis and visualized by ethidium bromide staining. **(E) **Densitometric analysis of data presented in (D) expressed as the ratio of CD109 PCR product to GAPDH internal control (CD109/GAPDH) is shown (*P *= 0.717).

The above results suggest that SSc fibroblasts express higher levels of CD109 protein *in vivo *compared with their normal counterparts and that SSc fibroblasts continue to express elevated CD109 protein *in vitro*. We further examined CD109 protein expression *in vitro *in SSc skin fibroblasts and normal skin fibroblasts by western blot (Figure 2A). Results shown in Figure 2A demonstrate that SSc skin fibroblasts show higher CD109 protein levels compared with normal skin fibroblasts. Densitometric analysis of CD109 protein levels (180 kDa band) in the cell lysates of SSc and normal skin fibroblasts was then performed using β-actin as a loading control. Results from two independent experiments corresponding to skin fibroblasts from two different randomly selected groups of SSc patients versus normal subjects are shown in Figure 2B. Further analysis of these data obtained from 16 SSc patients (nine with limited SSc and seven with diffuse SSc) and nine normal healthy controls was performed after reorganization into normal control, limited SSc and diffuse SSc groups. One data point (outlier) from the control group (value = 1.04) was removed. Determination of the fold-change in the ratio of CD109/β-actin in the limited SSc and diffuse SSc groups compared with normal controls shows that both limited SSc and diffuse SSc skin fibroblasts display significantly (*P *< 0.05) higher CD109 protein levels compared with normal skin fibroblasts (Figure 2C).

We next determined CD109 mRNA expression in SSc (*n *= 7) and normal (*n *= 7) skin fibroblasts by RT-PCR. Figure 2D indicates that SSc and normal skin fibroblasts display similar levels of CD109 mRNA expression (top panel). RT-PCR of GAPDH indicates that similar amounts of GAPDH are present in all samples (Figure 2D, bottom panel). Densitometric analysis of the data shown in Figure 2D is presented in Figure 2E and indicates that CD109/GAPDH levels in SSc and normal skin fibroblasts are not significantly different (*P *= 0.717). We further analyzed CD109 mRNA expression levels by real-time RT-PCR in SSc (*n *= 4) and normal (*n *= 3) skin fibroblasts and obtained similar results (data not shown).

### Treatment with exogenous TGF-β_1 _does not alter CD109 protein levels in SSc and normal skin fibroblasts

**Figure 3:**
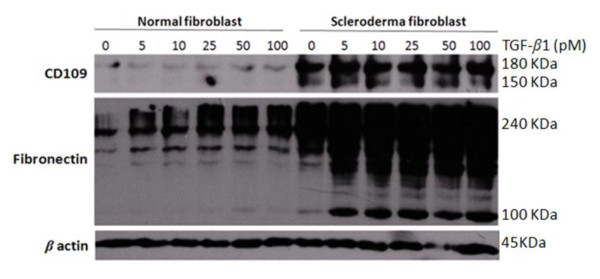
**CD109 protein expression is not regulated by transforming growth factor beta in systemic sclerosis and normal fibroblasts**. Systemic sclerosis (SSc) and normal skin fibroblasts were treated for 24 hours with 0, 5, 10, 25, 50 and 100 pM transforming growth factor beta 1(TGF-β_1_) Cell lysates were prepared and analyzed by western blot using anti-CD109 (top panel) and anti-fibronectin (middle panel) antibodies. The membrane was reprobed with an anti-β-actin antibody (bottom panel) to confirm that equal amounts of protein were loaded in each lane. Results presented are representative of three independent experiments. The representative SSc fibroblast data shown correspond to SSc Patient S2.

TGF-β is the most potent profibrotic cytokine known and has been implicated in the pathogenesis of SSc fibrosis [2,5]. Because our results indicate that CD109 protein levels are elevated in cultured SSc fibroblasts, we sought to determine whether TGF-β regulates CD109 protein levels. Figure 3 (top panel) shows that CD109 protein levels are higher in SSc skin fibroblasts compared with normal skin fibroblasts in the absence of TGF-β treatment (lane 1 vs. lane 7), as expected. Importantly, TGF-β treatment has no effect on CD109 protein levels in both SSc and normal skin fibroblasts (Figure 3, top panel). The responsiveness of SSc and normal fibroblasts to exogenous TGF-β is demonstrated by their increased fibronectin protein production (Figure 3, middle panel). Reprobing the membrane with an anti-β-actin antibody confirms that equal amounts of protein were loaded in each lane (Figure 3, bottom panel).

### CD109 inhibits ECM and CCN2 protein production in SSc and normal skin fibroblasts

**Figure 4:**
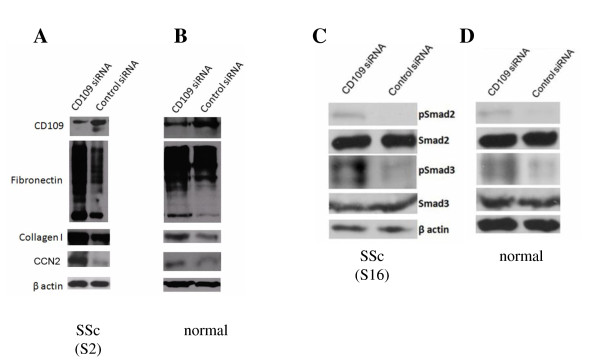
**CD109 inhibits extracellular matrix protein production and Smad2/3 phosphorylation in systemic sclerosis and normal fibroblasts**. **(A, C) **Systemic sclerosis (SSc) skin fibroblasts and **(B, D) **normal skin fibroblasts were transiently transfected with CD109-specific siRNA or control siRNA. Cell lysates were analyzed by western blot using (A, B) anti-CD109 (top panel), anti-fibronectin (second panel), anti-type I collagen (third panel) or anti-CCN2 (fourth panel) antibodies or (C, D) anti-phosphoSmad2, anti-phosphoSmad3, anti-Smad2 or anti-Smad3 antibodies. Membranes were reprobed with an anti-β-actin (bottom panel) antibody to confirm that equal amounts of protein were loaded in each lane. Results presented are representative of three independent experiments. The representative SSc fibroblast data shown correspond to (A) SSc Patient S2 and (C) SSc Patient S16.

It is well documented that SSc skin fibroblasts in culture continue to express elevated levels of ECM proteins such as fibronectin and collagen type I [15]. We therefore examined whether CD109 regulates levels of these ECM proteins in SSc skin fibroblasts by blocking CD109 expression using siRNA, followed by western blot analysis. Figure 4A shows that SSc skin fibroblasts transfected with CD109-specific siRNA display a marked reduction in CD109 protein level compared with control siRNA-transfected cells, as expected (top panel). Importantly, CD109 siRNA-transfected SSc skin fibroblasts display elevated fibronectin, collagen type I and CCN2 protein levels compared with control siRNA-transfected cells (Figure 4A, middle panels). Reprobing the membrane with an anti-β-actin (Figure 4A, bottom panel) antibody confirms that equal amounts of protein were loaded in each lane. Similar results are obtained using normal skin fibroblasts (Figure 4B).

### CD109 inhibits phosphorylation of Smad2 and Smad3 in SSc and normal skin fibroblasts

We have previously shown that CD109 inhibits Smad2/3 phosphorylation in human keratinocytes and other cell types [37]. Because ECM and CCN2 proteins are regulated by Smad-dependent mechanisms [27] we examined whether CD109 regulates Smad2/3 phosphorylation in SSc skin fibroblasts. Figure 4C shows that SSc skin fibroblasts transfected with CD109 siRNA display elevated Smad2 phosphorylation (top panel) and Smad3 phosphorylation (third panel) compared with control siRNA-transfected cells. Total Smad2 (second panel) and Smad3 (fourth panel) levels were not affected by CD109 siRNA knockdown (Figure 4C, middle panels). Reprobing the membrane with an anti-β-actin antibody confirms that equal amounts of protein were loaded in each lane (Figure 4C, bottom panel). Similar results were obtained using normal skin fibroblasts (Figure 4D). Although previous studies have reported elevated Smad2 and Smad3 phosphorylation in diffuse SSc fibroblasts [43,44], our results indicate that limited SSc and normal skin fibroblasts have similar Smad2 and Smad3 phosphorylation levels (Figure 4C, D), which might be attributed to phenotypic differences in TGF-β pathway activation between limited SSc and diffuse SSc skin fibroblasts [45,46].

### Soluble recombinant CD109 protein inhibits ECM and CCN2 production in SSc fibroblasts

**Figure 5:**
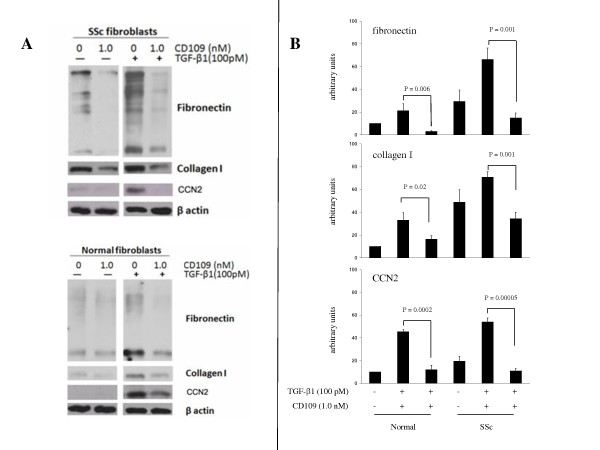
**Recombinant soluble CD109 protein inhibits transforming growth factor beta 1-induced extracellular matrix and CCN2 production**. **(A) **Systemic sclerosis (SSc) and normal skin fibroblasts were left untreated (-) or treated (+) for 24 hours with 100 pM transforming growth factor beta 1 (TGF-β_1_) in the presence of 1.0 nM recombinant CD109 protein or PBS/0.1% BSA vehicle control. Cell lysates were prepared and analyzed by western blot using anti-fibronectin (top panel), anti-type I collagen (second panel) or anti-CCN2 (third panel) antibodies. Membranes were reprobed with an anti-β-actin (bottom panel) antibody to confirm that equal amounts of protein were loaded in each lane. Results presented are representative of three independent experiments. **(B) **Densitometric analysis of the data from the above blot was performed using β-actin as a loading control. The representative SSc fibroblast data shown correspond to (A) SSc Patient S21 and (B) SSc Patients S19, S2 and S16.

We have previously shown that CD109 is released from the human keratinocyte cell surface [42], indicating that endogenous CD109 exists as both membrane-tethered and soluble forms. Our previous results also demonstrate that endogenous CD109 protein released from the cell surface and soluble recombinant CD109 protein both bind TGF-β_1 _with high affinity and inhibit TGF-β_1 _binding to its signaling receptors ([42] and unpublished observations, AB and AP, 2012). We therefore examined whether soluble recombinant CD109 protein inhibits TGF-β_1_-induced ECM and CCN2 protein production in SSc fibroblasts. Figure 5A shows that TGF-β_1 _induces fibronectin, collagen type I and CCN2 protein expression in SSc and normal skin fibroblasts as expected. Importantly, addition of recombinant CD109 protein leads to a marked reduction in TGF-β_1_-induced fibronectin, collagen type I and CCN2 protein expression in these cells. Reprobing the membrane with an anti-β-actin antibody confirms that equal amounts of protein were loaded in each lane (Figure 5A, bottom panel). Densitometric analysis of data obtained from three different experiments confirms that addition of recombinant CD109 protein results in a significant decrease in the production of TGF-β_1_-induced fibronectin, collagen type I and CCN2 in limited SSc and normal skin fibroblasts (Figure 5B). The results also indicate that limited SSc skin fibroblasts display higher fibronectin and collagen type I protein levels, but similar CCN2 levels, compared with normal skin fibroblasts under basal conditions.

## Discussion

SSc is a rare connective tissue disease of unknown etiology characterized by excessive ECM deposition in the skin and internal organs [2,6,13]. Studies over the past decade aimed at delineating the molecular mechanisms involved in SSc fibrosis have identified TGF-β as a central player and the TGF-β signaling pathway as an important target for therapeutic intervention for SSc [2,5,14]. We have previously identified CD109 as a TGF-β co-receptor that inhibits TGF-β signaling in human keratinocytes [37]. In the current study, we examine CD109 expression and function in limited SSc and diffuse SSc skin and normal skin *in vivo*, and in SSc and normal dermal fibroblasts and epidermal keratinocytes *in vitro*. Our results show that CD109 protein levels are elevated *in vivo *in both the dermis and epidermis of SSc skin compared with normal skin and *in vitro *in SSc dermal fibroblasts and epidermal keratinocytes compared with normal fibroblasts and keratinocytes, respectively. Although cultured SSc skin fibroblasts show higher CD109 protein levels compared with normal skin fibroblasts, they do not appear to display elevated CD109 mRNA levels. Elevated CD109 protein levels were observed in the skin tissue and fibroblasts from patients with both limited SSc and diffuse SSc, suggesting that increased CD109 protein expression might be a general feature of SSc. We also demonstrate that treatment with exogenous TGF-β_1 _does not alter CD109 protein levels in SSc or normal skin fibroblasts, suggesting that CD109 is not a direct target of TGF-β in these cells. Moreover, we found that CD109 inhibits ECM (fibronectin and collagen type I) and CCN2 protein production in both SSc and normal skin fibroblasts - as evidenced by the findings that CD109 siRNA-transfected SSc and normal skin fibroblasts display elevated fibronectin, collagen type I and CCN2 protein levels, whereas treatment with a recombinant CD109 protein leads to a decrease in levels of these proteins. In addition, we show that CD109 inhibits phosphorylation of Smad2 and Smad3 in SSc and normal fibroblasts. Taken together, this study identifies CD109 as a novel protein overexpressed in limited SSc and diffuse SSc skin fibroblasts and indicates that CD109 inhibits Smad2/3 signaling and ECM/CCN2 production in these cells, as in normal skin fibroblasts.

TGF-β co-receptors including endoglin, betaglycan and CD109 are important regulators of TGF-β signaling, and recent evidence indicates that they may modulate fibrotic gene expression in SSc fibroblasts. Endoglin expression has been reported to be higher in diffuse SSc skin fibroblasts compared with normal fibroblasts, and endoglin expression levels were shown to increase with disease progression [47]. A more recent study demonstrated that endoglin levels correlate with profibrotic marker (collagen I and CCN2) levels in diffuse SSc skin fibroblasts and that endoglin promotes profibrogenic Smad1 signaling in these cells [48]. In addition, cell surface endoglin and betaglycan expression levels were shown to be increased in diffuse SSc skin fibroblasts compared with normal fibroblasts and overexpression of betaglycan was sufficient to promote basal and TGF-β-induced CCN2 promoter activity in a mouse fibroblast cell line (NIH3T3) [49]. Our results showing that CD109 protein levels are elevated in both limited SSc and diffuse SSc skin tissue and cultured fibroblasts compared with controls suggest that upregulation of CD109 in these SSc subtypes may involve a common pathophysiological mechanism. Further studies to determine the expression and function of TGF-β co-receptors in the different clinical subsets of SSc, in particular their relevance to TGF-β-driven profibrogenic Smad1 signaling, is an important avenue for future research.

An important question raised from our results is why CD109 protein levels but not mRNA levels are increased in SSc fibroblasts. Our data showing that the levels of CD109 protein, but not of mRNA, are higher in limited SSc and diffuse SSc skin fibroblasts compared with normal skin fibroblasts suggests that post-transcriptional regulation of CD109 may be altered in limited SSc and diffuse SSc skin. Such alteration may involve enhanced CD109 protein synthesis and/or decreased CD109 protein degradation. Our preliminary results demonstrating that CD109 protein degradation is impaired in SSc fibroblasts (AB and AP, 2012, unpublished results) suggest that impaired CD109 degradation may be responsible for the elevated CD109 protein levels in SSc fibroblasts. It is interesting to note in this regard that several studies have reported increased TGF-β receptor protein levels in SSc fibroblasts [21,50], leading to the suggestion that TGF-β receptor stability is increased in SSc due to impairment of TGF-β receptor degradation [44]. Furthermore, recent microarray data [46,51] support our finding that CD109 mRNA levels in SSc and normal fibroblasts are similar. However, quantitative PCR analysis of RNA from a higher number of limited SSc and diffuse SSc dermal fibroblasts with age-matched, sex-matched and gender-matched control samples will be required to confirm these results.

Paradoxically, despite the elevated CD109 protein levels in SSc skin fibroblasts and the demonstrated ability of endogenous CD109 protein to inhibit ECM and CCN2 production, the levels of ECM and CCN2 in SSc fibroblasts remain elevated. One possible explanation for these results is that the upregulation of CD109 in SSc fibroblasts is not sufficient to completely counteract the activation of TGF-β or other profibrotic pathways in SSc. This notion is supported by our finding that addition of a recombinant CD109 protein to SSc fibroblasts, which already express elevated CD109 protein levels, is still able to decrease ECM and CCN2 levels. Whether the upregulation of CD109 is an adaptive response or a consequence of aberrant TGF-β signaling in SSc remains to be determined. Our results showing that CD109 is not a direct target of TGF-β in SSc and normal skin fibroblasts suggests that CD109 upregulation in SSc fibroblasts may involve TGF-β-independent mechanisms.

The most promising finding in the current study is that, despite the already upregulated CD109 protein expression in SSc, the addition of further exogenous CD109 is able to downregulate ECM production. Aberrant activation of autocrine TGF-β signaling is a critical factor involved in the maintenance of the fibrotic phenotype of SSc fibroblasts [16]. Identifying factors that inhibit TGF-β signaling in fibroblasts may thus lead to the development of novel anti-TGF-β therapies for the treatment of SSc. Our results showing that CD109 siRNA transfection leads to a marked increase, and that addition of recombinant CD109 protein results in a significant decrease, in the production of fibronectin, collagen type I and CCN2 in both SSc and normal skin fibroblasts suggest that CD109 has potent antifibrotic effects in these cells. In addition, our finding that CD109 siRNA knockdown in SSc and normal skin fibroblasts is associated with enhanced Smad2/3 phosphorylation suggests that endogenous CD109 inhibits autocrine TGF-β signaling in these cells. These results suggest that mechanism by which CD109 decreases ECM and CCN2 production in SSc and normal skin fibroblasts may involve inhibition of Smad2/3 phosphorylation. Together, our results suggest that recombinant CD109 protein is a promising candidate for antifibrotic therapy in SSc.

In addition to playing a possible pathophysiological role in SSc, the elevated CD109 protein levels may serve as a biomarker of disease activity in SSc. CD109 is a member of the α_2_-macroglobulin/complement family of thioester-containing proteins and has been shown to be expressed in endothelial cells, platelets and activated T cells [28,29]. Recent studies have shown that CD109 is differentially expressed in a variety of human tumors [30-34,37,52,53], leading to the suggestion that CD109 may represent a novel biomarker for certain cancers. The relatively small number of samples examined in the current study does not allow us to correlate CD109 expression levels with disease severity and/or duration. More work is needed to determine whether CD109 levels are differentially expressed at specific phases of disease activity in limited SSC and diffuse SSc.

## Conclusions

Despite the findings that CD109 is upregulated in SSc skin and cultured SSc skin fibroblasts, and that blocking CD109 expression in SSc cells leads to further production of ECM, it is clear that the CD109 response in SSc is insufficient to inhibit the production of excess ECM. Importantly, however, exposure of SSc fibroblasts to additional exogenous CD109 does result in the downregulation of excess ECM production - it is therefore worthwhile pursuing the role of CD109 as an antifibrotic agent and exploring whether CD109 protein expression represents a biomarker for active disease in SSc.

## Abbreviations

bp: base pair; CCN2: connective tissue growth factor; DMEM: Dulbecco's modified Eagle's medium; ECM: extracellular matrix; GAPDH: glyceraldehyde 3-phosphate dehydrogenase; PBS: phosphate-buffered saline; PCR: polymerase chain reaction; RT: reverse transcriptase; SSc: systemic sclerosis; siRNA: small interfering RNA; TGF-β: transforming growth factor beta.

## Competing interests

The authors declare that they have no competing interests.

## Authors' contributions

X-YM contributed to conception and design, acquisition of data, analysis and interpretation of data and drafting the manuscript. KWF contributed to conception and design, analysis and interpretation of data and drafting the manuscript. MB contributed to collection of samples and analysis and interpretation of data. AP contributed to conception and design, analysis and interpretation of data and drafting the manuscript. All authors contributed to revising the manuscript critically for important intellectual content, and read and approved the manuscript for publication.
